# Tobacco in Hysteria and Spasmodic Stricture of the Uterus

**Published:** 1844-03

**Authors:** Wm. B. Diver

**Affiliations:** Cincinnati


*Tobacco in Hysteria and Spasmodic Stricture of the Uterus.*—By Wm. B. Diver, M.D., of Cincinnati. In the American Journal of Medical Science, April, 1842, is recorded, a case of Hysteria cured by tobacco. On reference to my note-book, I found recorded, Philadelphia, Dec. 9th, 1841, a case of Hysteria, in which tobacco was used with the most prompt and beneficial effects. The patient was a servant in a highly respectable family in Philadelphia.

I was called to see her, at first, in a very distressing condition from having swallowed a number of large pins, which I succeeded, after a great deal of trouble, in removing from the œsophagus, with Dr. Bond's admirable Gullet Forceps.

Several weeks after the occurrence of this accident, I was again called to see her in violent hysterical convulsions. The spasmodic contractions were so strong as to require the united efforts of four powerful adults to prevent her being injured. The eyes were forcibly drawn towards the inner canthus, so as to present a case of double squinting. The pupils were contracted to a small point, and the iris was insensible to a brilliant light. The tongue was frequently protruded between the teeth, and severely wounded.

After considerable trouble I succeeded in opening a vein in the arm, and abstracted about 16 ounces of blood. This was followed by a temporary cessation of the convulsive throes, which, however, returned with increased violence, so that the patient was almost entirely unmanageable.

To prevent further injury of the tongue, and to facilitate the administration of medicine, a cork enveloped in the end of a towel was held between the teeth. Large doses of tinct. opii. and tinct. assafœtid. were administered, and attended with but transient effects; the convulsions were returning again with increased violence. In this stage of the case, finding the ordinary antispasmodics unavailing, I thought of using tobacco. I accordingly ordered a poultice of strong Scotch snuff to be applied warm to the epigastrium. Very soon after this application was made, the spasms began to decrease in frequency and violence; the countenance to assume a natural appearance, and after six hours of intense suffering, the patient became quiet and rational.

Several months elapsed before there was any return of the symptoms; then, however, the patient was removed from my observation, and treated with the ordinary remedies.

The utility of tobacco in *Spasmodic Stricture of the Urethra*, was forcibly exemplified in a case, several years before the one just related, occurred.

H. E., a respectable mechanic in Philadelphia, after indulging in venereal excess, found himself unable to void his urine. In the course of 24 hours the bladder became distended, presenting the elastic tumor above the pubis, which is so characteristic of this condition of things.

After ineffectual attempts to force the stricture with a gum-elastic catheter, and in the absence of a silver one, I applied wet tobacco leaves to the inguinal and femoral regions, with the most satisfactory result. The patient soon began to exhibit the peculiar effects of tobacco on the system: and in a little while the spasm became relaxed, the contents of the distended viscus discharged, and the sufferer relieved.

In order to prevent a recurrence of symptoms, a catheter was introduced and secured in the bladder by an appropriate bandage.

If any apology is due for bringing these cases before the profession, it may be found in the maxim—"*Palmam qui meruit ferat.*"—*Western Lancet*, January, 1844.

